# Detection of florfenicol resistance in opportunistic *Acinetobacter* spp. infections in rural Thailand

**DOI:** 10.3389/fmicb.2024.1368813

**Published:** 2024-05-03

**Authors:** Bernice Siu Yan Tan, Lalit Mohan, Wanitda Watthanaworawit, Thundon Ngamprasertchai, Francois H. Nosten, Clare Ling, Pablo Bifani

**Affiliations:** ^1^A^*^STAR Infectious Diseases Labs (A^*^IDL), Agency for Science, Technology and Research (A^*^STAR), Singapore, Singapore; ^2^Infectious Diseases Translational Research Programme, Department of Microbiology and Immunology, Yong Loo Lin School of Medicine, National University of Singapore, Singapore, Singapore; ^3^Shoklo Malaria Research Unit, Mahidol-Oxford Tropical Medicine Research Unit, Faculty of Tropical Medicine, Mahidol University, Mae Sot, Tak, Thailand; ^4^Department of Clinical Tropical Medicine, Faculty of Tropical Medicine, Mahidol University, Bangkok, Thailand; ^5^Centre for Tropical Medicine and Global Health, Nuffield Department of Medicine Research Building, University of Oxford, Oxford, United Kingdom; ^6^Lee Kong Chian School of Medicine, Nanyang Technological University, Singapore, Singapore; ^7^Department of Infection Biology, Faculty of Infectious and Tropical Diseases, London School of Hygiene and Tropical Medicine, London, United Kingdom

**Keywords:** florfenicol resistance, *floR*, *Acinetobacter* spp., chloramphenicol, horizontal gene transfer

## Abstract

Florfenicol (Ff) is an antimicrobial agent belonging to the class amphenicol used for the treatment of bacterial infections in livestock, poultry, and aquaculture (animal farming). It inhibits protein synthesis. Ff is an analog of chloramphenicol, an amphenicol compound on the WHO essential medicine list that is used for the treatment of human infections. Due to the extensive usage of Ff in animal farming, zoonotic pathogens have developed resistance to this antimicrobial agent. There are numerous reports of resistance genes from organisms infecting or colonizing animals found in human pathogens, suggesting a possible exchange of genetic materials. One of these genes is *floR*, a gene that encodes for an efflux pump that removes Ff from bacterial cells, conferring resistance against amphenicol, and is often associated with mobile genetic elements and other resistant determinants. In this study, we analyzed bacterial isolates recovered in rural Thailand from patients and environmental samples collected for disease monitoring. Whole genome sequencing was carried out for all the samples collected. Speciation and genome annotation was performed revealing the presence of the *floR* gene in the bacterial genome. The minimum inhibitory concentration (MIC) was determined for Ff and chloramphenicol. Chromosomal and phylogenetic analyses were performed to investigate the acquisition pattern of the *floR* gene. The presence of a conserved *floR* gene in unrelated *Acinetobacter spp*. isolated from human bacterial infections and environmental samples was observed, suggesting multiple and independent inter-species genetic exchange of drug-resistant determinants. The *floR* was found to be in the variable region containing various mobile genetic elements and other antibiotic resistance determinants; however, no evidence of HGT could be found. The *floR* gene identified in this study is chromosomal for all isolates. The study highlights a plausible impact of antimicrobials used in veterinary settings on human health. Ff shares cross-resistance with chloramphenicol, which is still in use in several countries. Furthermore, by selecting for *floR*-resistance genes, we may be selecting for and facilitating the zoonotic and reverse zoonotic exchange of other flanking resistance markers between human and animal pathogens or commensals with detrimental public health consequences.

## 1 Introduction

Florfenicol (Ff) is a synthetic broad-spectrum antimicrobial agent widely employed in veterinary medicine (Wu et al., 2021). Classified as an amphenicol, it irreversibly binds to the peptidyl transferase center of the 50S ribosomal subunit of prokaryotes. This binding inhibits the elongation of the peptide chain, ultimately hindering protein synthesis. Ff is primarily bacteriostatic in Enterobacteriaceae and *Staphylococcus aureus* (Wei et al., 2016; Somogyi et al., 2023; Trif et al., 2023; Guo et al., 2024) and bactericidal at clinical concentrations against *Haemophilus influnzae, Streptococcus suis, Mannheimia haemolytica*, and *Pasteurella multocida* (Graham et al., 1988; Illambas et al., 2013; Lei et al., 2018; Somogyi et al., 2023). Ff was granted approval by the Food and Drug Administration (FDA) for the treatment of bovine respiratory infections caused by pathogens such as *Pasteurella multocida* (White et al., 2000) and by the European Medicines Agency for the control of respiratory tract infections of bacterial origin in cattle and pigs (Kehrenberg and Schwarz, 2006). Unfortunately, the extensive usage of florfenicol in veterinary practice has led to a notable increase in resistance among zoonotic pathogens to this antimicrobial (White et al., 2000; Wasyl et al., 2013; Zhan et al., 2019; Kerek et al., 2023).

*Acinetobacter* is an opportunistic pathogen in humans and is often overlooked as a veterinary pathogen (Wareth et al., 2019). Its natural habitat is the environment, particularly soil and water. The bacterium is implicated in both community- and healthcare-acquired infections (Villalón et al., 2019; Wareth et al., 2019). Among the *Acinetobacter* species, *Acinetobacter baumannii* is the most clinically relevant species, causing most of the infections. Recently, other members of ACB complex members including *Acinetobacter pittii, Acinetobacter nosocomialis, Acinetobacter seifertii*, and *Acinetobacter lactucae* have also been isolated from patients (Migliaccio et al., 2023). These species cause infection in patients with comorbidities such as chronic lung disease, impaired immunity, malignancy, advanced age, diabetes mellitus, or renal diseases (Mancilla-Rojano et al., 2020).

The widespread and increased use of florfenicol in livestock, aquaculture, and poultry (Zeng et al., 2019; Guo et al., 2024) has accelerated the rate at which pathogens develop resistance to it (Zhan et al., 2019; Yang et al., 2022; Trif et al., 2023). The consumption of florfenicol in Europe falls under the category of class “C” (Caution) (Categorisation of Antibiotics Used in Animals Promotes Responsible Use to protect Public and Animal Health and European Medicines Agency, 2024). Florfenicol is widely used and, in some circumstances, reported to account for as much as 24.0% and 24.2% of all class C antimicrobials used during the weaning and fattening phases in pig rearing (Trevisi et al., 2022). Unsurprisingly, determinants of florfenicol resistance have been found in the environment associated with pig farms or in freshwater aquaculture (Fernández-Alarcón et al., 2010; Zhao et al., 2016; Zeng et al., 2019; Li et al., 2020; Fu et al., 2022; Wang et al., 2022; Lin et al., 2023). Several Ff resistance mechanisms have been identified, with the *floR* gene playing a significant role in conferring resistance. The *floR* gene encodes the FloR protein (with 12 hydrophobic transmembrane regions), which forms a proton motive force (PMF)-driven efflux pump that removes both Ff and chloramphenicol from bacterial cells using active transport (Adesoji and Call, 2020; Li et al., 2020). The presence of the *floR* gene has been reported in various genomes. For instance, Ff resistance has been associated with the presence of the *floR* gene on a transferable plasmid in *Klebsiella pneumoniae* in clinical isolates from China (Lu et al., 2018). The presence of *floR* variants (*floR-T1* and *floR-T2*) has been reported in the multidrug resistance (MDR) region as an integrative and conjugative element (ICE) in *Pseudomonas aeruginosa* (Qian et al., 2021). *Escherichia coli* isolates from clinical samples revealed the presence of the *floR* gene in transposon-like fragments with recombination-related genes along with *tetA* and *tetR* genes, which regulate tetracycline resistance (Møller et al., 2016; Lu et al., 2018). The occurrence of *floR* in drug-resistant regions on chromosomes with IS*91* family transposase in *Proteus vulgaris* has been reported (Li et al., 2020). The presence of the *floR* gene flanked by insertion sequences and other resistance genes in clinical isolates of *A. baumannii* has also been reported (Wareth et al., 2021; Zafer et al., 2021). These observations highlight a possible horizontal gene transfer-mediated zoonotic transmission of resistance genes associated with Ff resistance to human pathogens. High Ff resistance has furthermore been observed in environmental samples, as exemplified by Ff resistance isolated from four *Acinetobacter* spp. reported from water samples in Nigeria (Adesoji and Call, 2020).

In this study, we used multiple genetic and bioinformatic approaches to investigate the presence of the *floR* gene in *Acinetobacter* spp. isolates of human origin and the environment to identify the genes associated with the *floR* resistance cassette and to further demonstrate the potential transmission of resistance determinants to human pathogens and environmental microbes. The diversity of the strains carrying the *floR* gene suggests the occurrence of multiple transmission events leading to amphenicol resistance.

## 2 Materials and methods

### 2.1 Bacterial isolate collection

During a study conducted over 10 years (from 2009 to 2019), a total of 39 patients from three separate rural clinics in Thailand (Supplementary material) had isolated events of *Acinetobacter* spp. infections. The majority (34/39, 87.2%) of the bacterial isolates were isolated from blood samples of the infected patients. Furthermore, 5.1% (2/39) were isolated from cerebrospinal fluid samples (CSF), 5.1% (2/39) from urine samples, and 2.5% (1/39) from a urinary catheter. In addition to the clinical samples, 10 environmental samples were also collected from the same geographic area for environmental testing for infection prevention control (IPC), i.e., to contribute to the prevention of healthcare-associated infections (HAIs) through the detection of environmental contamination with key drug-resistant pathogens. Testing was carried out at one of the clinics to detect and monitor environmental contamination with target drug-resistant pathogens. Routine diagnostic media and culture conditions were used to isolate *Acinetobacter* spp. from clinical specimens, which included the use of Biomerieux BacT/Alert blood culture bottles for blood samples, Oxoid Brilliance™ UTI Clarity™ agar for urine samples, and CHROMagar ESBL for environmental samples.

### 2.2 Whole-genome sequencing and identification

DNA extraction was performed using the BioBasic One-4-All Genomic DNA Miniprep kit (cat: BS88503). The extracted DNA was sent to NovogeneAIT Genomics Singapore Pte Ltd. for whole genome sequencing (WGS). The isolates were sequenced using 2 × 151 bp paired-end reads on an Illumina HiSeq 4000 platform. The quality of the raw reads was analyzed using FastQC. The paired reads were assembled using SPAdes (v3.6.0) (Bankevich et al., 2012). Automatic annotation was carried out using RAST (Aziz et al., 2008).

### 2.3 Bioinformatic analysis

Speciation of the bacterial isolates was performed using average nucleotide identity (ANI). The genomes of the isolates were compared to the annotated *Acinetobacter* sequences available in GenBank. Species allocation was done with a limit of 95% identity to the annotated sequence (Jain et al., 2018). Abricate ResFinder was used to search for acquired resistance genes in the genome (Zankari et al., 2012). The web version of SimpleSynteny (Veltri et al., 2016) was used to construct the synteny plots. Circle plots were constructed using CIRCOS software using Command Line (Krzywinski et al., 2009).

### 2.4 Antibiotic susceptibility

The minimum inhibitory concentration (MIC_99_) values for Ff and chloramphenicol were determined according to the Clinical and Laboratory Standards Institute (CLSI) 2023 broth dilution guidelines, as the *floR* gene is also known to confer resistance against chloramphenicol (Bolton et al., 1999). According to the CLSI guidelines, a growth of ≥32 μg/mL of chloramphenicol is considered to be a chloramphenicol-resistant strain for non-*Enterobacteriaceae*. A growth of ≥ 8 μg/mL of Ff is considered as the Ff-resistant strain (Verner-Jeffreys et al., 2017). ASP 6 (*Acinetobacter variabilis*) served as a negative control for the MIC.

## 3 Results and discussion

Whole-genome sequencing (WGS) confirmed that all the isolates belonged to the genus *Acinetobacter*. The acquired resistance gene annotation of the isolates revealed that 10 out of the 39 clinical isolates (25.6%) and 2 out of the 10 (20%) environmental isolates harbored the *floR* gene in the bacterial genome (Table 1). Among the clinical isolates of *Acinetobacter* spp., 10% (1/10) of the isolated *A. baumannii*, 25% (1/4) of the isolated *A. pittii*, 60% (3/5) of the isolated *A. nosocomialis*, 40% (4/10) of the isolated *A. variabilis*, 25% (1/4) of the isolated *Acinetobacter junii*, and 100% (1/1) of the isolated *Acinetobacter johnsonii* were found to be harboring the *floR* gene. Among the environmental isolates of *Acinetobacter* spp., 100% (1/1) of the isolated *Acinetobacter schindleri* and 50% (1/2) of the isolated *A. variabilis* isolated were found to be harboring the *floR* gene. Through antibiotic susceptibility testing, we confirmed that all the clinical strains were resistant to florfenicol and chloramphenicol. The two environmental samples were borderline resistant to Ff and were either susceptible (ASP3) or intermediate (ASP18) to chloramphenicol (Table 1). Interestingly, ASP3 also encodes *cmlA1*, an additional chloramphenicol-resistant genetic marker (Li et al., 2014), while remaining susceptible to chloramphenicol. The susceptibility profiles were confirmed by independent biological replicates. An analysis to understand this conflicting observation for ASP3 is underway and is not part of this study. The Ff resistance due to the presence of the *floR* gene is responsible for cross-resistance to chloramphenicol (Zafer et al., 2021). The use of chloramphenicol is restricted in high-income countries, but it is still a drug of choice in many low- and middle-income countries for ophthalmic use and other infections (WHO Model List of Essential Medicines−22nd list, 2021, 2021). Thus, the spread of the *floR* gene will alter the use of chloramphenicol in many low- and middle-income countries.

**Table 1:** *floR*-positive isolates from 2009 to 2019 and their MIC_99_ values for florfenicol and chloramphenicol.

**Year of isolation**	**Sample ID and genome accession**	**Specimen type**	***Acinetobacter* spp**.	**MIC**_**99**_			
				**Florfenicol (**μ**g/mL)**		**Chloramphenicol (**μ**g/mL)**	
2011	ASP7 (JAZHCP000000000)	Blood	*Acinetobacter pittii*	≥40	R	≥80	R
2011	ASP2 (JAZHCO000000000)	Blood	*Acinetobacter variabilis*	≤ 20	R	≥80	R
2012	ASP12 (JAYXHY000000000)	Blood	*Acinetobacter variabilis*	≤ 20	R	≥80	R
2013	ASP16 (JAYXHZ000000000)	Urine	*Acinetobacter baumannii*	≥80	R	≥160	R
2013	ASP17 (JAYXIA000000000)	Cerebrospinal fluid	*Acinetobacter variabilis*	≥20	R	≥80	R
2015	ASP29 (JAYXIB000000000)	Blood	*Acinetobacter junii*	≤ 20	R	≥160	R
2016	ASP32 (JAYXIC000000000)	Blood	*Acinetobacter nosocomialis*	≥40	R	≥160	R
2017	ASP39 (JAYXID000000000)	Blood	*Acinetobacter nosocomialis*	≥40	R	≥80	R
2017	ASP36 (JAZHCN000000000)	Blood	*Acinetobacter johnsonii*	≤ 20	R	≥80	R
2017	ASP40 (JAYXIE000000000)	Blood	*Acinetobacter nosocomialis*	≥40	R	≥80	R
NA	ASP3 (JAYXIF000000000)	Environment	*Acinetobacter variabilis*	≥8	R	≤ 16	S
NA	ASP18 (JAYXIG000000000)	Environment	*Acinetobacter schindleri*	≥16	R	≥16	I
2011 (negative control)	ASP6 (JAZHCM000000000)	Blood culture	*Acinetobacter variabilis*	< 1	S	≤ 8	S

**Figure 1:**
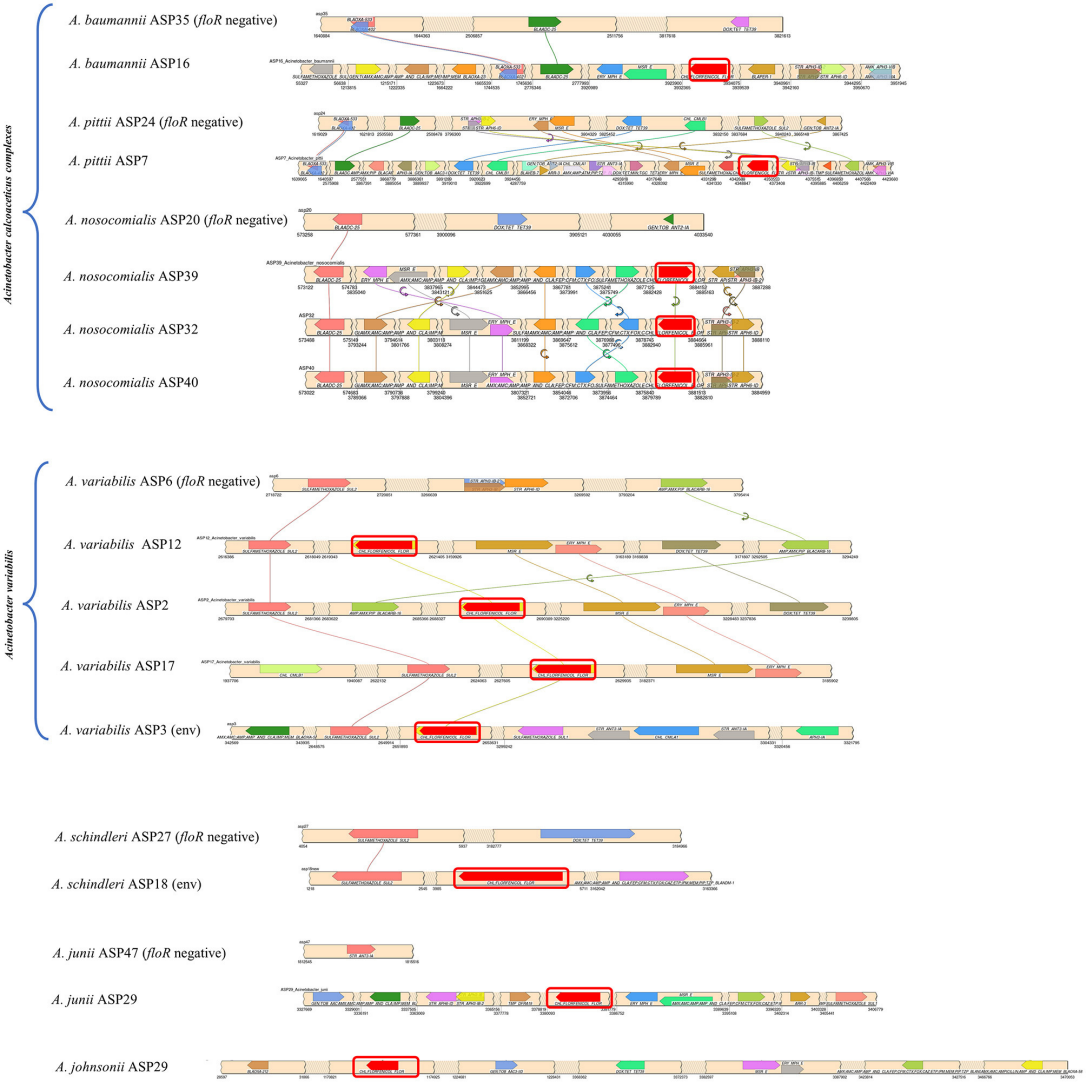
Synteny plot of the *floR* gene and associated acquired antibiotic resistance gene across the chromosome. The *floR* gene is highlighted with a red box. The values at the bottom of the beige ribbons represent the chromosomal location of the genes. Each *floR*-positive sample has been compared to the corresponding *floR*-negative strain from the study cohort, with the exception of ASP29, for which no corresponding counterpart was available within the cohort. (env): environmental isolates.

**Figure 2:**
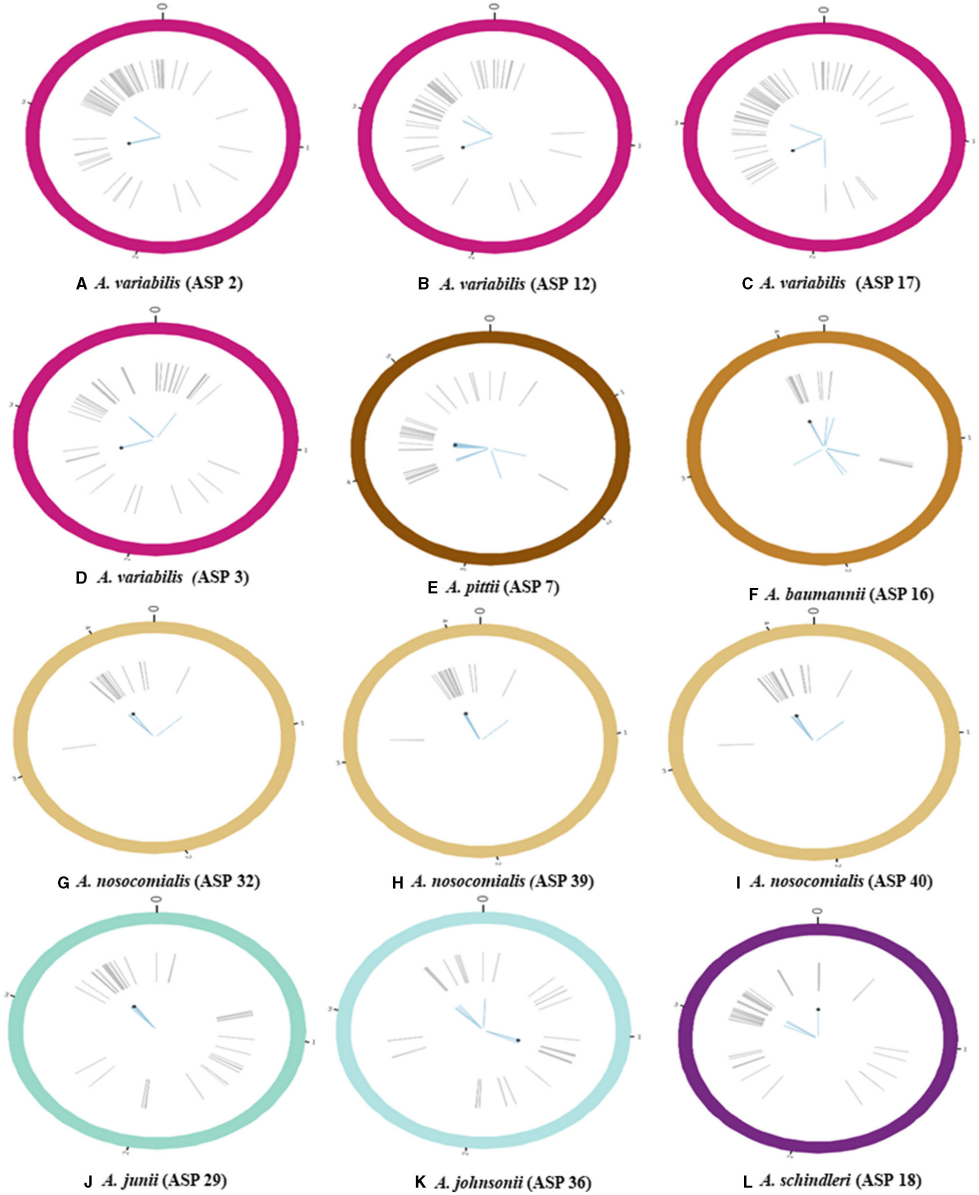
Schematic representation of bacterial chromosome harboring the gene conferring florfenicol (*floR*) resistance (all the values on the chromosomes are in mbp); **(A–D)** circle plots represent the bacterial chromosome of *A. variabilis*; **(E–I)** circle plots represent species belonging to the ACB complex [**(E)** *A. pittii*, **(F)** *A. baumannii*, and **(G–I)** *A. nosocomialis*]; **(J, K)** circle plots correspond to other *Acinetobacter* spp. [**(J)** *A. junii* and **(K)** *A*. *johnsonii*]; **(L)** the circle plot represents *A. schindleri*. Gray lines indicate the position of various mobile genetic elements (MGEs), blue-colored lines highlight annotated acquired antibiotic resistance genes, and the black point represents the location of *floR* on the chromosome.

**Figure 3:**
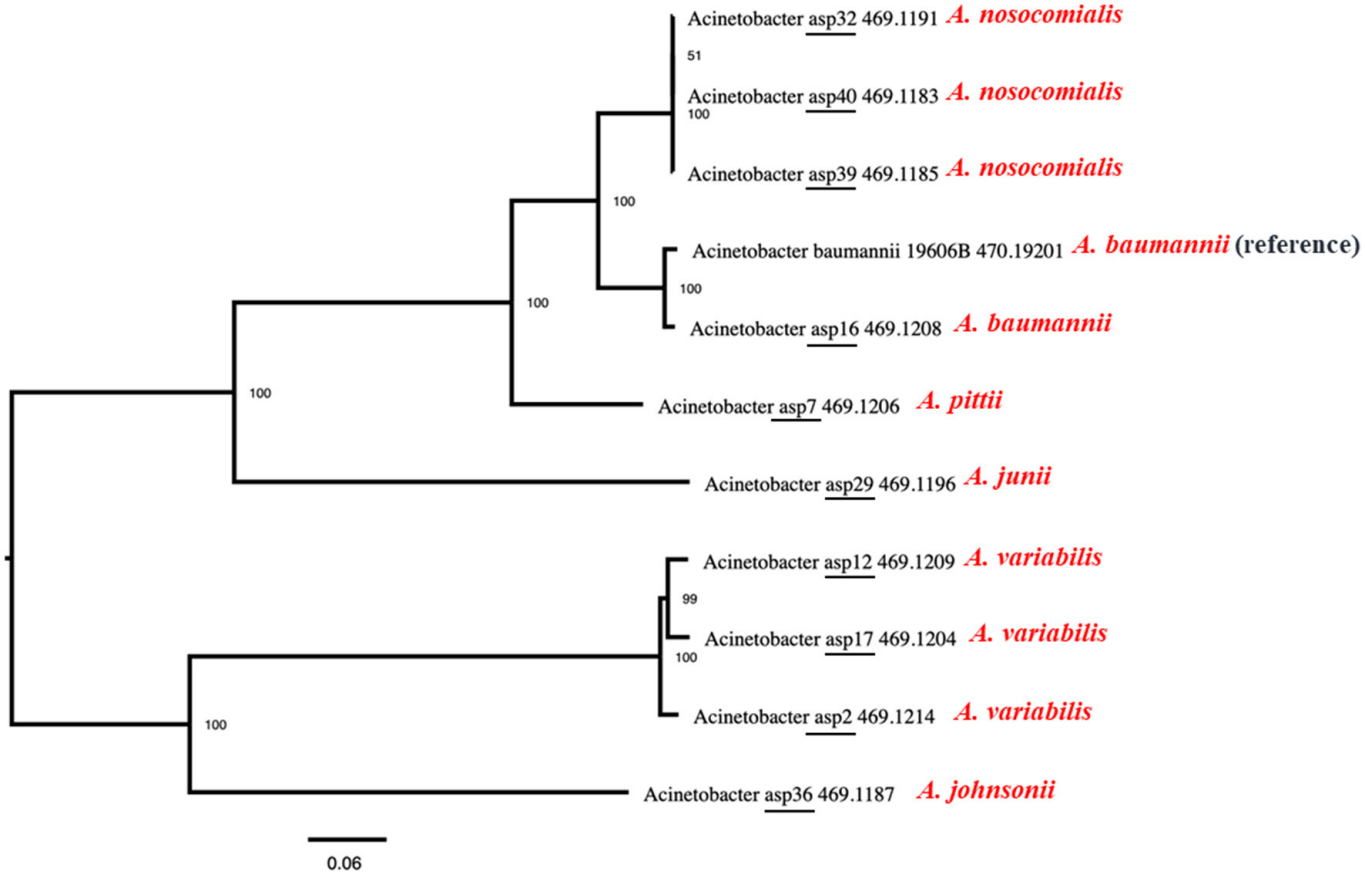
Maximum-likelihood phylogeny of the *Acinetobacter* species isolated in this study with the reference genome of ATCC *Acinetobacter baumannii* 19606. Average nucleotide identity was used to generate the phylogenetic tree, and the data show that the strains harboring the *floR* genes are highly diverse, with the exception of the *A. nosocomialis* isolates, which form one cluster.

The identified *floR* gene from all 12 isolates was found to be 100% identical to the *floR* gene of *Vibrio cholerae* (NCBI Reference Sequence: NG_047869.1) through a basic local alignment search tool (BLAST). Among the *floR-positive* isolates, three samples from clinical isolates (ASP2, 12, and 17) and one sample from an environmental strain (ASP3) belonged to *A. variabilis*. Five isolates belonged to the *Acinetobacter–calcoaceticus–baumannii* (ACB) complex and one isolate each to *A. junii, A. johnsonii*, and *A. schindleri*. Through chromosomal analysis, we observed the same *floR* gene in all isolates, but the flanking regions differed from each other (Figure 1). In all isolates, the *floR* gene appeared to be clustered within the variable region rich in mobile genetic elements (MGEs), as depicted in the circle plots (Figure 2). The phylogenetic analysis (Figure 3) of the isolates showed that the strains harboring the *floR* genes were highly diverse, with the exception of the *A. nosocomialis* isolates, which formed one cluster. WGS and chromosome analysis, phylogeny, and speciation suggest that with the exception of *A. nosocomialis*, the acquisition of *floR* is an independent event as they are found in different *Acinetobacter* species.

Figure 1 represents the chromosomal location of the *floR* gene along with all the acquired resistance genes in comparison to the *floR* negative strains as reference strains. The insertion of the *floR* gene in *A. baumannii* is in a variable region rich in MGEs (Figure 2). The insertion is near the *bla* gene when compared to the *floR*-negative strain. This association of the insertion near the *bla* gene is also observed across the ACB complex isolates. Through circle and synteny plots, we can observe that the *floR* gene is not accompanied by conserved flanks; however, the *floR* gene was always present in the region rich in MGEs. Similarly, for *A. variabilis* isolates, the *floR* gene was inserted near the *sul2* gene when compared to the *floR*-negative isolate. Notably, for both the ACB complex, *A. variabilis*, and other isolates, the *floR* gene insertion was found within the highly variable region.

The genomic annotation of WGS revealed that *floR* was present in recombination hotspots in all the isolates, accompanied by various MGEs, including insertion sequences (IS), transposons (Tn), and others (Figure 2). These recombination hotspots, also present in the *floR*-negative reference strains, harbor various other antibiotic resistance markers and prophage regions, indicating multiple events of gene uptake and a high frequency of recombination.

It is hard to determine a clonal link as multiple recombinations, insertions, and deletions might have occurred prior to and/or after the acquisition of *floR*. The isolates are phylogenetically far apart, the transfer of the *floR* gene is highly unlikely to have been occurred during speciation. Notably, all clinical samples have the macrolide resistance cassette (*msrE* and *mphE*) and the sulfamethoxazole resistance gene (*sul2*) in the proximity of *floR* in highly variable regions of the genome. Clinical isolates of *A. variabilis* harbor additional erythromycin and streptomycin B resistance genes (*msrE*) and erythromycin and macrolide resistance genes (*ery* and *mphE*). Furthermore, the *floR-*positive *A. variabilis* also harbors tetracycline (TET) and doxycycline (DOX) resistance genes. The environmental samples have the sulfamethoxazole resistance gene (*sul2*) located near the *floR* gene in both strains. Overall, WGS analysis and speciation alone suggest that, in the sample collection of this study, the acquisition of the *floR* gene is an independent event. It is highly possible that its selection and transmission is associated with the HGT of other drug-resistant markers. Other studies have also suggested similar possibilities (Kehrenberg et al., 2006; Verner-Jeffreys et al., 2017), but, as in this study, those inferences lack solid evidence.

## 4 Conclusion

*Acinetobacter* spp. isolates from humans and the environment encode the *floR* gene, which confers resistance to both florfenicol and chloramphenicol. Chloramphenicol-resistant *A. baumannii* (XDRAB), encoding the *cmlA1* gene, has been previously reported from Thailand. Chloramphenicol resistance was found to be widely prevalent in the non-target organisms in aquaculture environments in Southeast Asia in one study, but the mechanism of resistance was not reported (Huys et al., 2007; Chopjitt et al., 2020). In this study, speciation of the bacterial isolates using average nucleotide identity (ANI), combined with phylogenetic analysis, indicates that the strains reported are not epidemiologically related and, yet, they all carry the *floR* gene, suggesting the occurrence of independent events leading to the acquisition of the resistant markers. In all instances, the *floR* gene was found within variable regions, as depicted in the synteny plots. We elucidate that the wide use of florfenicol in livestock, poultry, and aquaculture production may be a driver for florfenicol resistance in veterinary and human pathogens. The consumption of florfenicol in animal farming has rapidly increased since its introduction (Wang et al., 2019). The presence of *floR* in both clinical and environmental samples indicates that the *floR* gene is persistent in the environment and can be incorporated into the genomes of different species. In all isolates, *floR* was located in the variable region of the bacterial genome, flanked by multiple mobile elements that host many other resistance genes. Therefore, by imposing the selection of the *floR* gene through the use of florfenicol, along with the selection for chloramphenicol resistance, we may be facilitating the HGT of other flanking-resistant markers, virulence factors, or other genetic elements. Florfenicol resistance may also facilitate reverse zoonosis and the reversible transfer of drug-resistant markers to veterinary pathogens and commensals. Overall, the presence of the *floR* gene in human and environmental isolates indicates that there is an ongoing genetic exchange between zoonotic pathogens, human pathogens, and environmental microbiomes.

## Data availability statement

The data presented in the study are deposited in the NCBI GenBank repository, accession numbers JAYXHY000000000, JAYXHZ000000000, JAYXIA000000000, JAYXIB000000000, JAYXIC000000000, JAYXID000000000, JAYXIE000000000, JAYXIF000000000, JAYXIG000000000, JAZHCM000000000, JAZHCN000000000, JAZHCO000000000, JAZHCP000000000.

## Ethics statement

The study did not require approval by an Ethics Committee as individual patient data, nor clinical specimens, were used. Only anonymized stored isolates obtained from routine hospital laboratory procedures were used in the study.

## Author contributions

BT: Writing—original draft, Visualization, Project administration, Methodology, Investigation, Formal analysis, Data curation. LM: Writing—original draft, Visualization, Project administration, Methodology, Investigation, Formal analysis, Data curation. WW: Writing—review & editing, Data curation. TN: Writing—review & editing, Data curation. FN: Writing—review & editing, Data curation. CL: Writing—review & editing, Data curation. PB: Writing—review & editing, Validation, Supervision, Resources, Project administration, Funding acquisition, Conceptualization.
