# Supplementary material for: Detection of florfenicol resistance in opportunistic Acinetobacter spp. infections in rural Thailand
Source: Front Microbiol. 2024 May 3;15:1368813. doi: 10.3389/fmicb.2024.1368813 (PMC11099283; doi:10.3389/fmicb.2024.1368813)
Supplement: Supplementary file 1 [file Presentation_1.PPTX]

## Slide 1
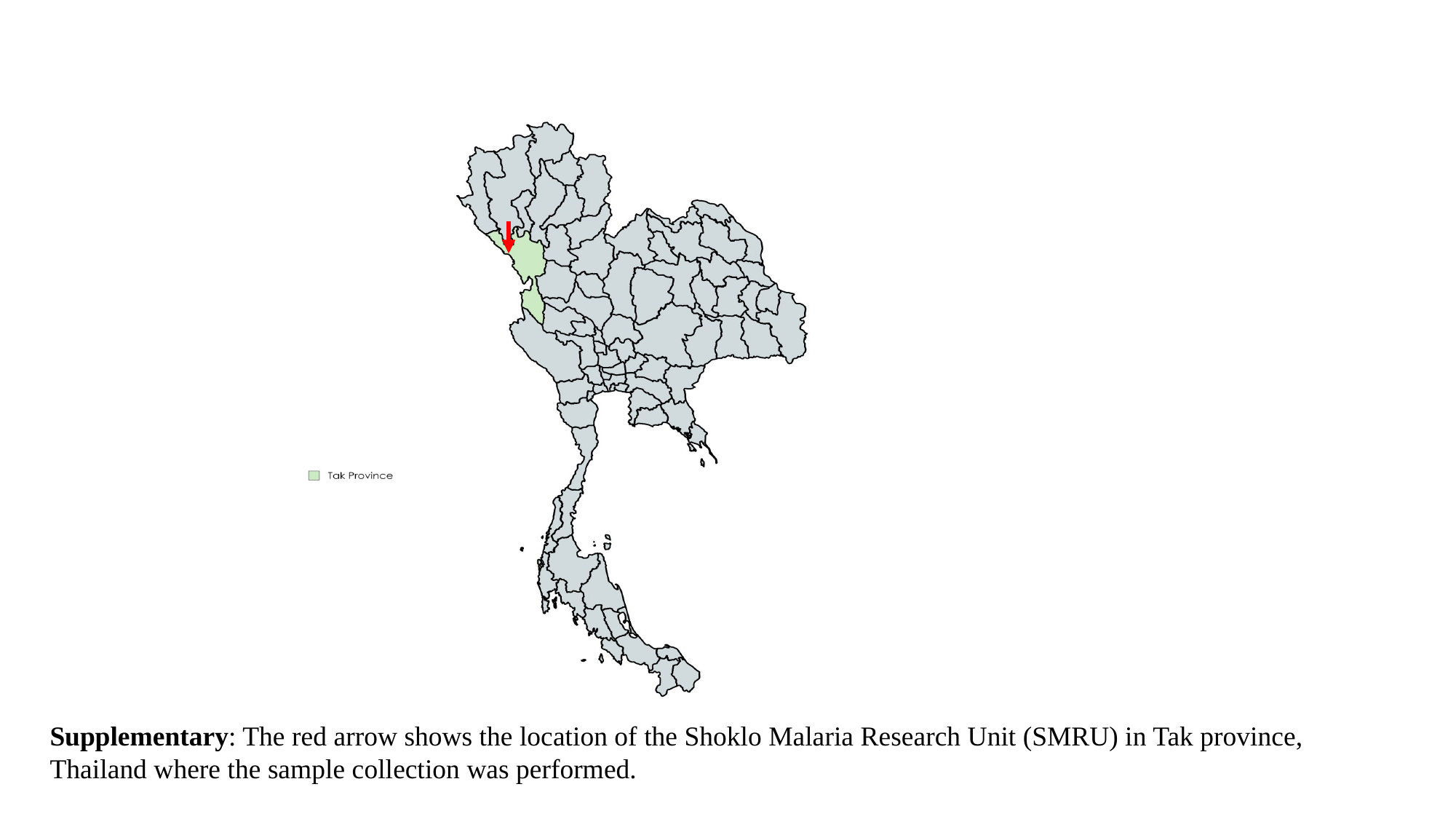

Supplementary: The red arrow shows the location of the Shoklo Malaria Research Unit (SMRU) in Tak province, Thailand where the sample collection was performed.
